# Control and guidance: a comparative study of building and planning standards for age-friendly built environment in the UK and China

**DOI:** 10.3389/fpubh.2023.1272624

**Published:** 2023-12-21

**Authors:** Fan Xie, Xiangfeng Li, Xiaoming Li, Zhulin Hou, Jing Bai

**Affiliations:** ^1^School of Architecture, Southeast University, Nanjing, China; ^2^Key Laboratory of Urban and Architectural Heritage Conservation of Ministry of Education, Southeast University, Nanjing, China; ^3^The Bartlett School of Architecture, University College London (UCL), London, United Kingdom

**Keywords:** control, guidance, United Kingdom, China, standards, age-friendly, comparative study

## Abstract

The crux of building and planning standards for age-friendly built environment in all countries resides in the regulation of age-friendly built environment practices, yet there exist variations in the representation of content dimensions. The UK is distinguished by its discretionary approach to guidance, whereas China exhibits a highly controlled disposition. Control and guidance may appear to be antithetical, it is essential to recognize that the building and planning standards for age-friendly built environment in both countries never deviate from the legal constraints while providing guidance in achieving age-friendly environments, thus striking a delicate balance between control and guidance. The study examines the standard systems of national standards, local standards and organizational standards, as well as the three dimensions of foundation standards, generic standards and specialized standards. The analysis of building and planning standards for age-friendly built environment in the UK and China scrutinizes the disparities between control and guidance, identifying similarities and differences in the building and planning standard system and content dimensions of the two countries. This analysis serves as a valuable reference for the development of building and planning standards for age-friendly built environment in China.

## 1 Introduction

The UK stands out as a country with a significant proportion of older population globally, and is considered highly advanced in terms of building and planning standards for age-friendly built environment. As early as the 1950s, the UK government began recognizing the significance of such standards due to the growing demand for older adult services. A series of building and planning standards for age-friendly built environment have been introduced successively to provide judicial solutions for the planning and implementation of age-friendly built environment. Although the older adult population in the UK has not experienced substantial growth since 1970, the process of aging has rapidly intensified (1), leading to the emergence of various standards targeting age-friendly built environment. With its extensive expertise in addressing the challenges of population aging, the UK provides valuable pragmatic insights in the development of standards in this field. Notably, the UK reached 7% proportion of older adults aged 65 and above, preceding China by a margin of 70 years.

China, on the other hand, accompanied by a continuous increase in its older adult population since 1999, when the proportion of older adults aged 65 and above reached 7%. By 2022, the overall population in China began showing negative growth after reaching its peak, presenting significant social challenges (2). In response, the Ministry of Housing and Construction issued Circular Jian Biao (2014) No. 23, which explicitly emphasizes the crucial role of engineering and land use standards as the technical foundation for constructing facilities dedicated to older adult services (3). Local authorities are tasked with further refining and augmenting national and industry standards, aiming to enhance their practicality and effectiveness. It is worth noting that China's development of a standard system for age-friendly built environment is still in the exploratory phase, constrained by factors such as planning, construction, service operation, and interdepartmental collaboration. Therefore, conducting a comparative analysis between China and the UK holds particular significance for China's endeavor to establish a comprehensive standard system for age-friendly built environment.

Researchers have been examining disparities between Asian economies and established institutional frameworks in developed nations, specifically in relation to housing and care provisions for the older adult (4, 5). Our efforts are dedicated to advancing public health services and promoting healthy aging through the study of building and planning standards for age-friendly built environment, regulation of aging-friendly construction practices, guidance on the creation of age-friendly built environments, and the facilitation of comprehensive public health initiatives. Moreover, our focus extends to the examination of whether and how the global trend of population aging has resulted in policy convergence between Eastern and Western economies, with the aim of identifying similarities and differences in standards systems. Despite varying dimensions, cultural contexts, and rates of aging, the UK and China exhibit a shared objective in the development and implementation of aging standards, namely, addressing the profound implications of population aging.

## 2 Processes of development

### 2.1 Development of building and planning standards for age-friendly built environment in the UK

The UK has played a pioneering role in establishing a comprehensive framework of building and planning standards for age-friendly built environment. The proportion of older adults aged 65 and above in the UK reached 7% as early as 1929. This demographic shift led to an increased demand for aging-related services across various aspects of social life, including housing, transportation, leisure, recreation, and care services. In response, the UK government sought institutional solutions to address these evolving needs.

**Figure 1:**
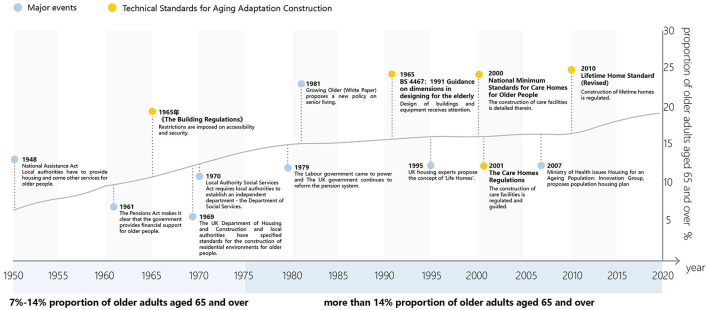
The evolution of age-friendly policies and standards in the UK.

Over time, a multitude of laws, regulations, strategic plans, and guidelines have been developed to promote age-friendly buildings and facilities (6), as depicted in Figure 1. Historical milestones include policy documents enacted before World War II, such as the Old Age Pensions Act of 1908 and the Poor Law Amendment Act of 1934, which primarily focused on ensuring livelihood security and sickness insurance for the older adult. Subsequently, the passage of the National Assistance Act in 1948 mandated that local authorities provide a range of services, including housing and medical care, for older individuals unable to support themselves (7).

The UK's building and planning standards for age-friendly built environment have witnessed continuous growth and refinement since then. Initially, the Building Regulations of 1965 imposed accessibility and safety requirements on new constructions. In 1969, the British Standards Institution published Anthropometric and Ergonomic Recommendations for Dimensions in Designing for the Elderly (BS 4467: 1969), providing anthropometric and ergonomic recommendations for designing spaces catering to the older adult. This standard was later updated in 1991 as Guide to Dimensions in Designing for Elderly People (BS 4467: 1991), which offered guidance on equipment and building design for older individuals. In the 21st century, additional standards such as The Care Homes Regulations 2001, National Minimum Standards for Care Homes for Older People, and the Lifetime Home Standard (Revised) further expanded the repertoire of building and planning standards for age-friendly built environment in the UK.

Undoubtedly, the UK has demonstrated a wealth of experience and an increasing abundance of standards in the realm of building and planning standards for age-friendly built environment, encompassing residential accommodations and facilities tailored to meet the needs of older individuals.

### 2.2 Development of building and planning standards for age-friendly built environment in China

**Figure 2:**
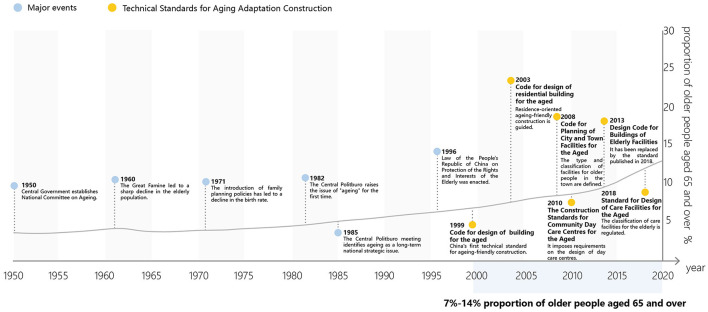
The evolution of age-friendly policies and standards in China.

As the proportion of older adults aged 65 and above in China reached 7% in 1999, the development of building and planning standards for age-friendly built environment commenced (Figure 2). The initial standard, Code for Design of Buildings for Elderly Persons (JGJ 122-99), issued in 1999, presented requirements pertaining to the design of environmental foundations, building structures, and building equipment for facilities catering to the older adult. This code addressed fundamental and universally applicable design needs, playing a pivotal role in the implementation of national policies and regulations concerning the older adult (8). Subsequent to this, Code for Design of Residential Building for the Aged (GB/T 50340-2003) was introduced in China in 2003. Serving as a cornerstone guideline, it provided a basis for constructing age-oriented residential buildings for older adults. In 2008, the Code for Planning of City and Town Facilities for the Aged (GB 50437-2007), was released and enforced nationwide. This code outlined detailed classifications and scalable indicators for urban facilities dedicated to the older adult (9). Additionally, in 2010, the Ministry of Housing and Urban-Rural Development and the National Development and Reform Commission approved the release of Construction Standards for Community Day Care Centers for the Aged (Jian Biao 143-2010) and Construction Standards for Elderly Care Homes (Jian Biao 144-2010), which addressed specific types of facilities for the older adult. These two standards prescribed requirements for site selection, planning layout, community size, area indicators, building design, and facility configuration (10, 11).

Regrettably, these standards were primarily guiding provisions at the macro level of design, placing more emphasis on area indicators and offering less comprehensive control over specific design implementations. Consequently, the efficacy of age-friendly built environment was somewhat limited. With the increasing demand for aging-related services (12), China's building and planning standards for age-friendly built environment have been subject to accelerated updates. Outdated standards cannot maintain their dominance. In 2013, the Ministry of Housing and Construction introduced the Design Code for Buildings of Elderly Facilities (GB 50867-2013). Subsequently, in 2016, the Code for the Design of Residential Buildings for the Aged (GB 50340-2016) was issued, only to be repealed two years later in 2018. Taking these revisions as a foundation, the Standard for Design of Care Facilities for the Aged (JGJ 450-2018) was promulgated in 2018. This updated standard focused on various types of facilities providing centralized care services for the older adult (13). Furthermore, in 2019, the Ministry of Housing and Construction implemented the Technical Standard for Intelligent Systems for Elderly Services (JGJ/T484-2019), signifying the era of intelligent older adult care in age-appropriate building and facility construction.

### 2.3 Analysis of development process

The development history of the UK and China shows that the UK and China started to gradually formulate building and planning standards for age-friendly built environment when the population aged 65 or above accounted for 7% of the total population. In the following 8–12 years, building and planning standards for age-friendly built environment were intensively introduced. The two countries have displayed proactive engagement in the exploration of spatial scales encompassing macro, meso and micro dimensions, primarily focusing on the city, community and building.

At the city scales, the UK has devised a comprehensive set of supportive literature pertaining to the creation of age-friendly urban environments. These documents encompass a range of aspects such as caregiving provisions, healthcare facilities, housing and community development, national housing strategies, as well as the formulation of inclusive design guidelines aimed at guiding the construction of age-friendly cities. For instance, within the National Planning Policy Framework, provisions have been established to account for age-friendly design principles in public transportation systems and the accessibility of publicly accessible spaces. On the other hand, China's approach is centered on addressing demands and resolving issues. The country has undertaken holistic planning efforts to determine objectives and key initiatives for age-friendly construction. Moreover, China has established standards and implementation regulations governing the establishment of ancillary facilities for older adult services in newly constructed urban areas, residential districts, as well as older urban regions. For instance, the Standard for Urban Residential Area Planning and Design (GB 50180-2018) has been revisited to incorporate additional standard provisions relating to the planning of residential areas in order to cater to the requirements associated with population aging.

At the community scales, building and planning standards for age-friendly built environment in the UK primarily center around the development of accessibility systems, recreational spaces, and community care facilities. Relevant guidelines provide instructive measures, with Building for Life 12 serving as a prominent example, urging local authorities, developers, and communities to forge thriving community locales predicated upon the guidelines. In contrast, China has implemented a system of home service facilities by leveraging the community as its building block within construction and planning standards aimed at fostering age-friendly built environments. By adhering to these standards, China aims to achieve comprehensive support coverage in newly constructed neighborhoods, while addressing shortcomings in established communities, thereby establishing convenient and entirely age-friendly communities. Notably, the Code for Accessibility Design (GB 50763-2012) expands the scope of accessibility implementation, placing particular emphasis on the design requirements that cater to the older adult population.

At the building scales, the UK classifies residential structures for the older adult into three main categories: all-age housing, older adult's housing, and care facilities. Among these, “older adult's housing” receives significant attention, with national strategies and local plans outlining corresponding objectives and requirements. The Department of Housing, Communities and Local Government (DCLG) in the UK, for instance, has released the Lifetime Homes Design Guide and Lifetime Homes Standard as guiding frameworks for nationwide development. These resources promote the integration of inclusive design principles, targeting diverse occupant needs throughout different stages of housing utilization. In China, there is a strong commitment to enhancing senior care services at various levels and establishing an aging-appropriate housing product system that caters to the diversified housing demands of the older adult population. Standards such as Design Code for Residential Buildings (GB 50096-2011) incorporate age-friendly adaptations to facilitate aging in place.

In summary, it is apparent that the building and planning standard systems for age-friendly built environment differ across various spatial scales in the two countries. These disparities are founded upon national policies, laws and regulations, which reflect the socioeconomic development levels in accordance with the theoretical principles guiding aging progression and the associated internal dynamics.

## 3 Comparison of building and planning standard systems for age-friendly built environment in the UK and China

### 3.1 National Standards

Standard-setting bodies in the UK encompass legislative, governmental, and third-party organizations. The Department of Housing, Communities & Local Government serves as the key governmental agency akin to China's Ministry of Housing and Urban-Rural Development. Its remit comprises care and service facilities (14), housing and community construction, as well as national housing strategies and plans. UK's national standards for age-friendly built environment prioritize the well-being and safety of older individuals, extending their influence into national legislation (15). Consequently, strategic planning at the national level harmonizes with legally binding standards, granting them judicious enforceability. Complementing these regulations, the UK government issues technical guidelines that furnish specific indicators and technical pathways. While the Building Regulations offer distinct objectives and legal constraints from a legal certainty perspective, they lack explicit technical indicators and control requirements, being more conceptual and general in nature (16). Conversely, the Approved Documents, which serve as accompanying technical guidelines, provide granular control and programmatic guidance concerning specific building targets. These standards adopt a hierarchical approach, ranging from overarching to detailed controls. For instance, concerning the design of accessible ramps, Appendix 1 of the Building Regulations stipulates that “Stairs, ladders and ramps shall be so designed, constructed and installed as to be safe for people moving between different levels in or about the building.” The Approved Documents outline specific implementation pathways, such as “If either a wheelchair user cannot see from one end of the ramp to the ramp has three flights or more then provide intermediate landings a minimum of 1800mm wide and a minimum of 1800 mm long as passing places.” Additional departments also contribute to building and planning standards for age-friendly built environment, including the issuance of National Minimum Standards for Care Homes for Older People by the UK Department of Health (17). The standard establish baseline requirements for services and environments within care facilities, for example, “Pre-existing homes that provide at least 4.0 sq. meters of indoor communal space for each resident as at time of implementation of these Standards shall continue to do so.” “Pre-existing care homes with rooms which provide 9 to 12 sq meters of useable space for each resident shall make 12 sq. meters available within a maximum of 10 years as of the date of implementation of these Standards.”

China's national standards encompass various aspects, including urban planning for older adult facilities, barrier-free facility design, community older adult facility layout, and age-appropriate housing design. In contrast to the UK's legal and regulatory framework, China's national standards primarily focus on control requirements at the indicator level. These standards provide specific construction indicators and standard design methods to ensure legal certainty and enable macro-regulation of age-appropriate housing construction by the central government. For instance, the Codes for Accessibility Design (GB 50763-2012) in China not only offers diverse design indicators for barrier-free ramp design but also provides standard sample drawings as construction references. This ensures orderly development in age-friendly built environment projects (18). The revision of the Standard for Urban Residential Area Planning and Design (GB 50180-2018), introduced provisions for residential area planning in response to the needs of aging and aging in place (19). This top-level design guidance plays a crucial role in facilitating the smooth implementation of age-friendly built environment. It is worth noting that these objectives align with the UK's national standard targets and share similarities in specific indicators. For example, both China and the UK specify that the slope of the ground at flat-sloped entrances for barrier-free ramps should not exceed a ratio of 1:20, demonstrating commonality in this aspect of their standards.

### 3.2 Local standards

Since the 1980s, the UK government has undergone a transition away from the welfare policies advocated by the Labor Party, leading to a consistent reduction in public welfare expenditure and the near complete privatization of older adult care services. Given this context, the UK government's stance is to avoid an excessive imposition of national binding standards concerning housing and care facilities targeted toward older individuals. Instead, local authorities and commercial developers have been afforded flexibility in designing and constructing homes tailored to the demands of the local market. Consequently, specific codes like the Building Better Care Homes for Adults and the Older People's Housing Design Guidance have emerged. Presently, in the UK government's efforts to establish building and planning standards for age-friendly built environment, considerations related to market dynamics and economic viability assume paramount significance. However, it is worth noting that market forces can perpetuate the potential for social exclusion among older segments of society (20). Although the central government assumes that local authorities will develop standards aligned with prevailing local practices, the majority of these authorities neither prioritize nor possess the capacity to effectively coordinate professional teams to facilitate this process (21). Consequently, only a handful of local authorities have formulated comprehensive standards for aging, while most areas still lack specific guidelines dedicated to promoting the construction of age-friendly housing. Thus, housing design for the older adult in the majority of regions primarily adheres to general housing design standards, supplemented by references to diverse design guidelines.

London, being the capital city, exemplifies an extensive repertoire of strategies employed by local government authorities to address the challenges associated with population aging. Each borough within London has established specific targets for addressing the needs of their respective aging populations. These targets, however, do not possess legal binding and are subject to the discretionary actions of each individual borough council. Notably, the Older People's Housing Design Guidance in the London Borough of Kensington puts forth a recommendation stipulating that “Within the bedrooms and lounges of residents' individual dwellings the bottom glazing line of a window should not be higher than 800 mm, and preferably not higher than 600 mm above finished floor level. Glazing below 800 mm must provide containment and guarding.” (22). It is important to emphasize that this provision functions exclusively as a guiding principle rather than an obligatory requirement governing the design of housing intended for older individuals. In a bid to ensure the alignment between local plans and demographic shifts along with market dynamics, London Councils actively foster the development of tailored strategic plans and standards by individual boroughs. However, it is worth noting that solely the Royal Borough of Kensington has taken the initiative to introduce design guidelines specifically targeting housing for older adults. While other boroughs have formulated certain policies pertaining to age-friendly built environment, the establishment of precise and focused standards remains elusive.

China's local standards are an extension of the nationally binding standards, encompassing provinces, autonomous regions, and municipalities directly under central government jurisdiction. Given China's vast size and varying natural and geographical factors across regions, engineering construction necessitates tailored technical measures and specific requirements based on local conditions and construction expertise (23). Encouragingly, pioneering regions in China that prioritize age-friendly built environment have proactively promoted the adoption of relevant local standards. These standards generally draw upon the guiding principles of national standards while considering the local level of aging development and the practical demands of age-friendly built environment (24).

To develop these standards, local governments in collaboration with relevant departments and think-tank experts conduct extensive research and expert consultations, aligning them with their specific regional development context. For instance, Beijing Municipality's Specification for Configuration of Facilities and Equipment of Senior Care Stations (DB11/T 1515-2018) mandates compliance with the relevant provisions stated in GB 50763, focusing on barrier-free facilities (25). Similarly, Shanghai's Design Standard of Building of Elderly Facilities stipulates that the floor area ratio for senior care facilities should not exceed 0.3 in new areas and 0.6 in central old urban areas (26). Notably, the requirement specifying that “the length of outdoor ramps should not exceed 12m, with a slope no steeper than 1/12,” demonstrates relatively more leniency compared to the national standard, which states that “the slope of the ground at flat entrances should not exceed 1:20.” Thus, the diversity in content among these local standards reflects a harmonious coexistence between overarching guidance at the national level and localized autonomy in shaping standards for age-friendly built environment in China.

### 3.3 Organizational standards

In addition to government-issued standards, the UK benefits from a wealth of third-party standards and research reports that serve as supporting documents. These encompass contributions from thematic research committees established by the UK Parliament or government, third-party research organizations, industry associations, and universities. These resources cover a diverse range of topics, providing detailed content and being published with greater frequency. Notably, they have exerted a significant influence on the advancement of building and planning standards for age-friendly built environment.

For instance, the Habinteg Housing Association published the Lifetime Home Standard (Revised), which has received official endorsement from the UK government (27). This standard places a stronger emphasis on the perspective of older individuals, featuring more comprehensive details and sub-categories compared to national and local standards. Moreover, it offers a wider variety of performance indicators and offers more guidance specific to different types of age-friendly built environment. As an illustrative example, one requirement specifies that “Adequate fixing and support for grab rails should be available at any location on all walls, within a height band of 300–1800 mm from the floor (28).” This particular directive addresses a concern not explicitly covered by national and local standards.

In China, the Program for Deepening Standardization Work Reform [Guo Fa (2015) No. 13] prescribes the relevant requirements followed by the AQSIQ and the National Standards Committee in formulating the Guidance on Fostering and Developing Organizational Standards. According to this guidance, social groups are authorized to develop organizational standards when national standards, industry standards, and local standards are absent. This enables them to swiftly address standardization needs driven by innovation and market demands, thereby filling gaps in existing standards (29). Notably, social groups are encouraged to establish organizational standards that are stricter than national and industry standards, thereby spearheading industry and enterprise development while enhancing product and service competitiveness in the market.

As part of the comprehensive standardization reform and in response to the evolving needs of the older adult population, China has made significant progress over the years in developing and releasing numerous volumes of social organizational standards. For example, the China Association for Engineering Construction Standardization has developed the Technical Standard for home-based Elderly Care Renovation of Urban Communities (T/CECS 1042-−2022), the Architectural Society of China has created the Standard for Design of Outdoor Healthy Environment for the Aged (T/ASC 18-2021), and the China Association of Social Welfare and Senior Service has produced the Facilities and Equipment Configuration for Pension Institutions (T/CASWSS 003-2019). Effective July 1, 2021, the Standard for Design of Outdoor Healthy Environment for the Aged (T/ASC 18-2021) enforces regulations regarding aspects of age-friendly environments not covered by national and local standards (30). As an illustration, it specifies “Set up planting beds of different heights, vertical greening and height-adjustable hanging flower baskets, etc. according to the physical condition of the older adult. For standing postures, the appropriate touchable height of plants is 850~1,650 mm, and for sitting postures, the appropriate touchable height of plants is 650~1,200 mm.”

In conclusion, a comparison between China and the UK reveals distinct perspectives and approaches in establishing a building and planning standard system, as illustrated in Table 1. Nevertheless, both countries build upon national policies, laws, and regulations, with an emphasis on technical aspects and product considerations within their respective national standards. Industries, localities, and social groups to further refine these standards and develop additional measures. Given the substantial demand for age-friendly built environment in China, drawing lessons from the UK's standards in this field can offer valuable insights for the overarching design of age-friendly built environment in China. Such knowledge transfer has the potential to contribute to the robust and sustainable development of age-friendly built environment practices within the country.

**Table 1:** Comparison of the UK's and Chinese building and planning standard systems for age-friendly built environment.

**Standard system**	**National standards**		**Local standards**		**Organizational standards**	
**Country**	**UK**	**China**	**UK**	**China**	**UK**	**China**
Subject of standard setting	Legislative branch, government ministries	Government ministries	Local government	Governments of provinces, autonomous regions and municipalities directly under the Central Government	Thematic research committee, third party research institute, industry association or university set up by the UK Parliament or Government	Academies, associations, chambers of commerce, federations, industrial technology unions and other social groups
Concept	Making standards binding at the judicial level	Guidance from a top-level design perspective	Develop standards based on local realities with encouragement from central government	Determine implementation with reference to the top-level design	A major influence on the development of standards for age-friendly built environment at national and local levels	Propose implementation rules/management practices based on government guidance
Role	Giving targets and legal limits for age-friendly built environment	Giving control requirements at the indicator level	Provides more guided construction methods	Enhanced control of national standards at the local level	Complementing national and local standards that are not covered	Responding to market demand and enhancing the competitiveness of our products and services

## 4 Comparison of three dimensions of building and planning standards for age-friendly built environment in the UK and China

China's existing standard system for age-friendly built environment encompasses a categorization of technical standards into three distinct dimensions: foundation standards, Generic standards, and specialized standards. This classification effectively serves the purpose of delineating various applicable objects and scopes within the field. Conversely, in the UK, the development of technical standards largely extends from its legislative and normative frameworks, with a key focus on accentuating the seamless coordination among design principles, humanistic care, and adherence to legislation and norms. In terms of the coordinated relationship between the content of the standards and the application characteristics of the British technical standards system basically conforms to the three dimensions of foundation standards, Generic standards, and specialized standards.

### 4.1 Foundation standards

The foundation standards provide a technical framework that serves as the underpinning for building and planning standards for age-friendly built environment, acting as a foundation upon which subsequent standards can be built (31). These fundamental standards delineate the mandatory criteria that must be met by all other standards within the same professional realm, ensuring compliance and coherence. Additionally, they encompass a wide range of elements including terminology, symbols, graphics, moduli, units, and various other standard types, contributing to a comprehensive and unified approach in the development and implementation of age-friendly built environment practices.

**Figure 3:**
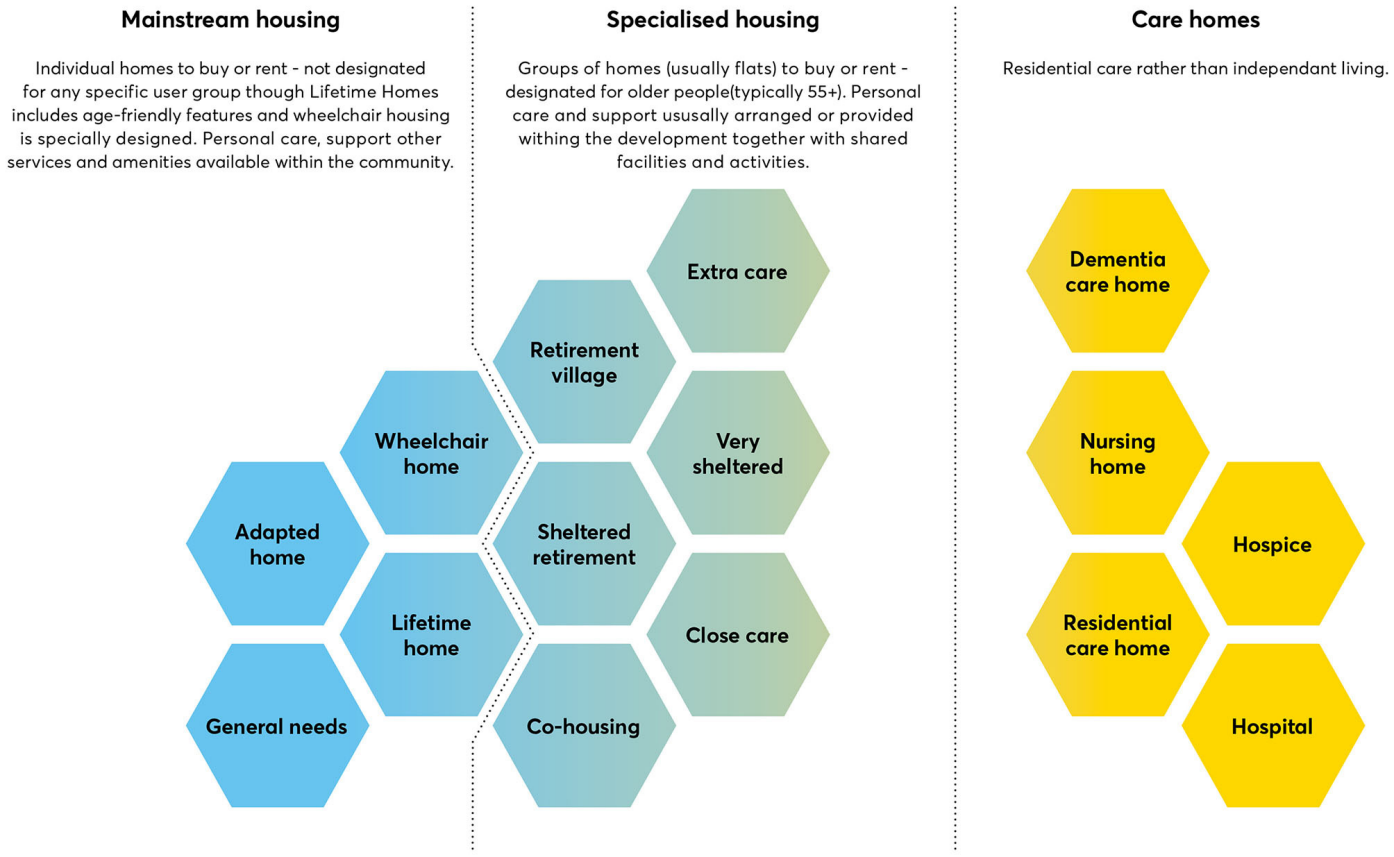
Types of buildings for older adults in the UK's building and planning standards system, Reproduced with the permission of ref. (32), copyright @ Center for London, 2021.

The foundation standards in the UK encompass a range of legislations and regulations pertaining to age-friendly built environment, providing definitive guidelines for the conceptual framework within this domain. For instance, these standards categorize buildings designed for older individuals into three main categories: lifetime homes (27), specialized homes, and care homes (Figure 3). In accordance with the use class in the Town and Country Planning System of the United Kingdom, all-age housing is designated as C3—dwelling houses, representing general housing. The classification of older adult housing has exhibited variation between C2 and C3, varying regionally and over time within the same region. Care homes fall under C2—residential institution, which encompasses nursing homes, care homes, and similar establishments. The classification of buildings and use class directly influences the standards applied in project approval, development, construction, and subsequent operation and management of diverse age-friendly structures, underscoring the significance of the foundation standards in the UK.

The foundation standards in China primarily aim to establish a standardized terminology and nomenclature system for age-friendly buildings and facilities, encompassing general provisions, general terms, and specialized terms. Within various standards pertaining to age-friendly built environment, these terms are defined; however, inconsistencies arise in the naming and definition of certain terminologies across different standards. For instance, in the Code for Design of Buildings for Elderly Persons (JGJ 122-99), a home for the older adult (nursing home) is defined as a social service institution providing comprehensive facilities for older adults to spend their old age peacefully, encompassing living arrangements, cultural and entertainment amenities, and healthcare services. In contrast, the Code for Design of Residential Building for the Aged (GB/T 50340-2003) released in 2003 define a nursing home as a collective residence for the older adult equipped with relatively complete supporting service facilities. Moreover, the latest Standard for Design of Care Facilities for the Aged (JGJ 450-2018) define nursing homes within the context of full day care facilities for the older adult. Consequently, the fundamental role of China's foundation standard lies in its guidance for overarching planning and coordination among disparate standards within the defined scope.

Similar issues regarding underlying standards also occur in both the UK and China. The UK experiences fluctuations in defining the types of older adult housing, while China encounters differences in the naming and definition of certain technical terms. These inconsistencies are attributed to varying legal interpretations in establishing standards for actual age-friendly built environment. Therefore, there is a pressing need for a “substantive determination” of terms within the standard system to harmonize concepts based on essential attributes. Consequently, this challenge must be addressed in the subsequent revision of standards in both countries.

### 4.2 Generic standards

Generic standards in the context of age-friendly built environment serve as significant indicators that reflect the commonalities found within other relevant standards (31). They provide a foundational framework upon which specialized standards can be formulated, particularly those encompassing vital aspects such as public safety, fire protection, energy conservation, environmental preservation, evaluation methodologies, and various other fields.

Generic standards pertaining to age-friendly built environment are incorporated into the control requirements for all buildings in the UK. These standards align with universal specification requirements (33) and classify the constituent elements of age-friendly built environment into distinct categories, establishing control targets within the parameters of the foundational standard. The generic standards also offer partially guided implementation recommendations, encompassing both objectives and means, while providing indicative construction measures aligned with the binding objectives. For instance, the UK's Approved Documents K mandate that “Stairs, ladders and ramps shall be so designed, constructed and installed as to be safe for people moving between different levels in or about the building.” It is further emphasized that “The standard of provision may need to be higher in a public building than in a dwelling, because people may not be familiar with the building and there may be more users.” As a result, specific construction standards have been proposed, such as “The maximum pitch for a private stair is 42°.” “For school buildings, the preferred going is 280 mm and rise is 150 mm.” The principle of universal and inclusive design constitutes a fundamental aspect of these generic standards, facilitating compatibility with diverse user groups and effectively meeting the varying requirements of different age-appropriate building types (34, 35).

The current generic standards in China adopt a region-specific approach, taking into account the average level of development in each area. Given China's extensive territorial expanse and varying demographics and economic conditions across regions, aligning the universal indicators with the requirements for age-friendly built environment presents challenges. Despite the inclination of older individuals toward inclusive design (36), achieving a harmonious fit between universal indicators and age-friendly built environment needs remains elusive across all regions. For instance, the Code for Planning of City and Town Facilities for the Aged (GB50437-2007) stipulates that the total number of beds allocated to nursing homes, older adult flats, and older adult care homes should be determined based on a range of 1.5–3.0 beds per 100 older adults. Currently, only select economically developed cities in China can fulfill this requirement, while less affluent cities and towns fall short with an average bed count of <1.0 per 100 older adults. Implementing such targeted age-friendly built environment objectives prove less attainable in economically disadvantaged areas. Consequently, the disparities stemming from regional unevenness in China give rise to a misalignment between supply and demand (37). Thus, comprehensively planning age-friendly built environment around the distinct needs of every older adult for an inhabitable environment becomes unviable, necessitating refinement in addressing this incongruity within the framework's revision and enhancement.

Both the UK's and Chinese generic standards exhibit a clear demarcation among building categories in the realm of age-friendly built environment, encompassing housing for the older adult as well as care facilities. At the crux of these standards lies an emphasis on generic target requirements, prevailing across both the Chinese and the UK's frameworks. However, the UK's generic standards furnish specific technical methodologies within predetermined objectives, while Chinese generic standards incorporate construction objectives within the purview of control requisites intertwined with limitations. Consequently, this discrepancy highlights a subtle divergence between the two sets of generic standards.

### 4.3 Specialized standards

Specialized standards pertain to the diverse subcategories of building and planning standards for age-friendly built environment (31). These standards possess a distinct and singular scope of application rendering them more precise and directed in nature. They effectively capture the specific attributes and necessities of particular professions or industries while also visually articulating the evolving iterations of standard technologies.

The specialized standards in the UK are characterized by a target-oriented and performance-driven approach. These standards adopt a results-focused perspective and offer some degree of autonomy in decision-making while maintaining control over the final outcome. Within the specific textual compositions of specialized standards, the UK standards typically commence with an overview of the core content and distinctive features of the standard, followed by a precise and controlled implementation strategy and measures. These standards encompass both qualitative descriptions and mandatory design constraints.

An exemplification of this can be seen in the National Minimum Standards for Care Homes for Older People which outline that the spatial requirements in personal accommodations should be suitable for individual user's use and specify that “In all new build, extensions and first time registrations, places provided as single rooms shall have a minimum of 12 sq. meters usable floor-space (excluding en-suite facilities).” It is noteworthy that the implementation measures often offer guiding principles and approaches, such as the recommendation that “Room dimensions and layout options shall ensure that there is room on either side of the bed, to enable access for carers and any equipment needed.” These provisions effectively ensure adaptive compliance with the specialized standards.

The specialized standards in China exhibit a heightened level of specificity with respect to the type of building, tailoring their content to meet the unique requirements of each specific building category. These standards are intricately linked to a particular building type or category, incorporating precise control measures that directly address the actual needs of users. For instance, the Construction Standard for Community Day Care Centers for the Aged (Jian Biao 143-2010) classifies community day care centers based on the size of the local population. It stipulates that “the building area of day care center housing for the older adult in communities categorized as first, second, and third, may be approved at respective ratios of 0.26 m^2^, 0.32 m^2^, and 0.39 m^2^ of building area per older adult.”

These building and planning standards for specific building types substantially enhance the significance and applicability of the specialized standards. On one hand, they serve to explicitly define the notion of diverse age-friendly buildings; while on the other hand, they put forth implementation specifications based on the actual construction and usage requirements associated with different building typologies.

The convergence of specialized standards between China and the UK exhibits a notable degree of coherence. The emergence of these standards is primarily driven by the imperative to address distinct categories of needs. Against the backdrop of a growing demand for customized age-appropriate products within the older adult market (38), the pervasive establishment of specialized standards represents an undeniable historical trend.

## 5 Control and guidance

As previously stated, the statutory UK standards pertaining to aging primarily take the form of general requirements with articulated objectives, accompanied by control requirements serving as guiding principles. The pursuit of actual user needs is underscored by these objectives, with standards being established through regulatory provisions and seeking institutional reliance. Notably, the incorporation of age-friendly built environment within the social welfare system introduces a political dimension to the normative nature of the standards. Within this framework, the control requirements outlined in the UK standards for age-friendly built environment offer a foundational benchmark for tangible implementation, addressing users' fundamental needs while allowing for further enhancements.

For instance, the National Minimum Standards for Care Homes for Older People stipulate that “In all newly built homes and first time registrations the home shall provide indoor sitting, recreational and dining space (referred to collectively as indoor communal space) apart from residents' private accommodation and excluding corridors, balconies and entrance hall amounting to at least 4.0 sq. meters for each resident.” By referencing controlled minimum standards, the implementation can meet the basic demands of users, while also accommodating local context and permitting flexible designs to facilitate effective execution.

To sum up, the UK standards for age-friendly built environment establish a balance between rigidity and flexibility, whereby the control requirements serve as guidelines empowering development in accordance with the degree of population aging.

In the context of China's national policies, the formulation of building and planning standards for age-friendly built environment follows a top-down approach from central guidelines to local implementation rules. In contrast to the UK, where comprehensive and statutory general requirements are established to guide the development of age-friendly built environment, China's standards primarily focus on regulating the process itself rather than providing explicit guidance for its development. Consequently, these standards do not effectively serve as a social welfare guarantee for the aging population.

While China's standards for age-friendly built environment are not lacking in terms of control measures when compared to the UK, they do exhibit a relative weakness in guiding the actual development of age-friendly infrastructure, particularly due to the national or local strategic planning objectives are often articulated in the guideline to promote the development of undertakings for the aged and not specified in the standards. Despite the lack of an explicit strategic intent, it is important to recognize that China's standards still play a pivotal role in shaping the trajectory of age-friendly built environment. The development of building and planning standards for age-friendly built environment in China is driven by overarching national policy guidelines, emphasizing their importance as a significant outcome of strategic planning.

This process operates indirectly, with the promulgation of standards necessitating compliance with professional specifications in the implementation of age-friendly built environment. Adherence to these specifications serves as a constraint on the practical implementation, while their long-term impact guides the overall direction and specific control aspects of age-friendly built environment. Simultaneously, the guiding elements of the strategic plan are influenced by robust control constraints.

Given the high level of compatibility between the standards and the strategic plan, age-friendly built environment in China is effectively guided by the strategic objectives embedded in the control provisions of these standards. Through this mechanism, political will translates into actionable guidance, which is encompassed within the control framework.

The UK standards for age-friendly built environment prioritize several key control points, including the clarity of planning, layout form, intensity, and the integration of vertical and horizontal links. These standards emphasize providing guidance during implementation, allowing for a significant degree of discretion to accommodate the changing needs associated with aging. This approach grants autonomy in making concrete implementation decisions.

In contrast, China's standards for age-friendly built environment exhibit a distinct focus on aligning the standards with strategic planning as a means of guidance. Consequently, these standards embody a dual attribute of both control and guidance, where the concept of guidance is encompassed within the framework of control. This approach ensures that fundamental requirements for age-friendly built environment are clearly defined while remaining adaptable to local conditions. It allows for flexible design, facilitates effective and tangible implementation, and guides age-friendly built environment to showcase the exemplary features of traditional Chinese culture.

The differing political, economic, and management systems between China and the UK provide a rationale for the divergent approaches taken in their respective standards for age-friendly built environment.

## 6 Conclusions

The study's findings reveal distinct characteristics in the approaches of the UK and China toward building and planning standards for age-friendly built environment. Firstly, the UK demonstrates a top-down guiding characteristic, evident across three levels: national standards, local standards, and organizational standards. In contrast, China exhibits a top-down controlling characteristic. Secondly, an analysis of the three dimensions of foundation standards, generic standards, and specialized standards reveals the presence of ambiguities in both countries. The UK's building and planning standards for age-friendly built environment exhibit a stronger focus on goal-oriented rational guidance. Conversely, China faces challenges stemming from the imbalanced regional development, leading to potential confusion regarding applicability. Thirdly, concerning the utility of building and planning standards in promoting age-friendly built environment, strong similarities exist between the two countries. Both the UK and China adopt dynamic top-down control and guidance mechanisms. However, variations in regulatory approaches translate into differing degrees of freedom during the actual implementation process. The UK benefits from greater flexibility in implementation, whereas China would benefit from bolstering inter-departmental collaboration. In a broader context, both the UK and China actively promote building and planning standards for age-friendly built environment through dynamic control and guidance mechanisms to enhance the aging-friendly renewal of physical environments, including living spaces and public health facilities, with the aim of promoting the development of age-friendly community environments. These initiatives are instrumental in addressing the health needs of older adult populations and play a critical role in elevating the overall age-friendly quality of living environments. Furthermore, concerted endeavors have been dedicated to embodying the prevailing social trend of upholding respect, compassion, and assistance toward the older adult, while continuously striving to bolster their sense of fulfillment, well-being, and safety.

## 7 Discussion and recommendations

The comprehensive analysis presented above reveals that the UK's standards for age-friendly built environment exhibit a higher degree of systematicity and hold significant reference value for China. Embracing lessons from the UK's standards can effectively contribute to the construction and advancement of building and planning standards for age-friendly built environment within China. This serves as a crucial point of reference for the development of technology standards in age-friendly built environment, particularly in finding an optimal equilibrium between control and guidance. Ultimately, this facilitates the creation of an age-friendly environment in China and fosters the sustainable development of an innovative health service system.

Through comparative analysis of building and planning standards for age-friendly built environment in China and the UK, it is evident that the creation of age-friendly built environments entails a complex and comprehensive system. It is especially imperative that environments are designed in an activity-friendly manner that also considers the needs of an aging population (39). This undertaking demands collaborative efforts from government entities, enterprises, social organizations, industry experts, and other stakeholders to engage in interdisciplinary and cross-disciplinary research, as well as to continually refine and enhance practical implementation processes. Timely amendments and refinements must be made throughout the implementation process, as different regions and sectors may have varying interpretations of some provisions in standards. This disparity may hinder achieving approval for design content and ultimately impact the design of age-friendly built environments. For example, the Standard for Design of Care Facilities for the Aged (JGJ 450-2018) introduced in 2018 includes a safety evacuation provision stating that “doors opened to the public activity area for the older adult should not impede transportation.” However, there is no clear explanation of what constitutes “public activity areas for the older adult,” resulting in significant discrepancies between design units and review departments and necessitating substantial plan revisions during the approval stage. These limitations and constraints can significantly impede implementation and coordination.

To achieve a harmonious equilibrium between control and guidance in China's building and planning standards for age-friendly built environments, the following recommendations are proposed:

### 7.1 Emphasizing systematicness and cohesiveness

Considering the institutional disparities between China and the UK, the control functions within China's aging standards are dispersed among various government departments due to differing administrative authorities. This fragmentation results in indicator requirements being segmented according to the perspectives of different departments, consequently confining the scope of the standards within the jurisdictional boundaries of these authority departments. In contrast, the UK employs a distinct legislative approach wherein all units formulate standards based on legislative requisites. As a result, the standard system established for age-friendly built environment is characterized by enhanced scientific rigor and systematicity.

Therefore, acquiring insights from the UK's methodology holds promise for cultivating a more comprehensive and coherent development of standards through separate legislation specifically targeting age-friendly parameters. Concurrently, this approach facilitates the establishment of an integrative management structure grounded in legislation. Concurrently, during the implementation phase, it is imperative to establish standardized indicators for performance evaluation to gauge housing demand and the compatibility of older adults with their living environment, thereby ensuring comprehensive and efficient resource utilization. Moreover, it is imperative for China's building and planning standards to elucidate the interface division and integration between the existing standard system and the prevailing national and industrial standards. Adhering to the principles of directly adopting quality standards, supplementarily revising standards requiring updates, and formulating new standards where gaps exist, a meticulous assessment and classification of individual standards should be conducted. By doing so, an integrated system of building and planning standards for age-friendly built environment can ultimately be consolidated.

### 7.2 Coordinating purposes and perspectives

In contrast to the UK's legislative approach, which addresses the issue of standard uniformity in age-friendly built environment at the statutory level, China's standards for age-friendly built environment exhibit a fragmented landscape, characterized by diverse competent units, formulating entities, and guiding perspectives. Consequently, there exist discrepancies in attribute definitions and instances of contradictions and duplication within certain index requirements across building and planning standards for age-friendly built environment.

Therefore, it is imperative to enhance the platform and mechanism for communication and consultation between the management units and formulating entities responsible for building and planning standards for age-friendly built environment. Building upon the existing standards, it becomes necessary to further delineate the management authority of age-friendly built environment, clarify the positioning and efficacy of various types of standards, and reinforce effective communication channels among different departments. This collaborative effort aims to foster coordination and minimize discordant factors among diverse sets of standards. Through promoting unity and complementarity in the content of the building and planning standard systems for age-friendly built environment, continuous improvement can be achieved in the overall standard framework.

### 7.3 Enhance adaptability and flexibility of standards for age-friendly built environment

Age-friendly built environment encompasses a broad spectrum of areas, each characterized by distinct needs and technical considerations. However, both China and the UK encounter challenges regarding the alignment of standards with the varying needs to a certain extent. In the UK, for instance, a multitude of products and ambiguously defined types of older adult housing (40) contribute to unclear enforceable laws, regulations, and construction service standards, consequently impacting project approvals, as well as overall development and construction endeavors. Similarly, individual age-friendly standards in China also lack well-defined typologies, necessitating unified and coordinated determinations from various governing departments.

Furthermore, China's current building and planning standards for age-friendly built environment exhibit a certain level of decision-making autonomy within the prescribed guidelines. However, this autonomy is often subjected to controlling requirements, thus falling short in accommodating all possible scenarios comprehensively. Unlike the UK's notable emphasis on a high degree of discretion, China should focus more on striking a balance between legal certainty and flexibility in its standards. Enhancing the market-oriented mechanism becomes imperative, necessitating effective guidance for enterprises, associations, and other third-party institutions to actively contribute. By harmonizing and enhancing the scope of control within the standards while concurrently providing support through comprehensive guidance content, diverse usage needs can be better accommodated in Chinese practices.

## Author contributions

FX: Writing – original draft, Writing – review & editing. XianL: Writing – review & editing. XiaoL: Writing – review & editing. ZH: Writing – original draft. JB: Writing – original draft.
